# Supplementary material for: The Consumption of Beeswax Alcohol (BWA, Raydel®) Improved Zebrafish Motion and Swimming Endurance by Protecting the Brain and Liver from Oxidative Stress Induced by 24 Weeks of Supplementation with High-Cholesterol and D-Galactose Diets: A Comparative Analysis Between BWA and Coenzyme Q10
Source: Antioxidants (Basel). 2024 Dec 5;13(12):1488. doi: 10.3390/antiox13121488 (PMC11672924; doi:10.3390/antiox13121488)
Supplement: Supplementary file 1 [file antioxidants-13-01488-s001.zip › Supplementaryantioxidants-3319387.pdf]

Supplementary Material

Supplementary Table S1

RAYDEL
Health Confidence

Raydel Australia Pty Ltd
Level 1, building 1, 9-15 Chilvers Rd.
Thornleigh NSW 2120 Australia
Tel +61 2 9480 1300
Fax +61 2 9480 1399
A.B.N. 45 054 555 903
www.raydel.com.au
info@raydel.com.au

Certificate of Analysis

Product name: Beeswax alcohols

Batch #: 330020123

Date of Manufacture: 17/01/2023

Parameter	Results	Approved Limits
Aspect	Powder	Powder
Color	White bone	Off white to cream
**Identity and Purity***		
1-tetracosanol (C_{24})	6.12 %	6-15 %
1-hexacosanol (C_{26})	10.72 %	7-20 %
1-octacosanol (C_{28})	13.75 %	12-20 %
1-triacontanol (C_{30})	30.52 %	25-35 %
1-dotriacontanol (C_{32})	22.08 %	18-25 %
1-tetratriacontanol (C_{34})	2.95 %	≤ 7.5 %
**Total (Purity*)**	86.14 %	**≥ 85 %**
**Other quality specifications**		
**Melting temperature**	80.1-81.6 °C	78.0 – 85.0 °C
**Loss on drying**	0.60 %	≤ 1.0 %
**Heavy metals (Pb, Cd, Hg)**	<0.0000115 %	≤ 0.001 %
**Residual solvents**		
**Acetone**	≤ 0.03	≤ 0.03 g/kg
**Hexane**	≤ 0.005	≤ 0.005 g/kg
**Microbiological content ****		
**Total Aerobic Microbial Count**	≤ 10	≤ 10^3 per g
**Yeast and mould**	≤ 10	≤ 10^2 per g
**Enterobacteria**	≤ 10	≤ 10^2 per g
***Staphylococcus aureus, Pseudomonas aeruginosa, Escherichia* coli, Candida albicans**	Absent	Absent in 1 g
Salmonella sp	Absent	Absent in 10 g
**Observations**		
**References:**	* Manufacturer GC validated method; purity expressed as the total of high molecular weight alcohols.	
	** BP	

Note about storage conditions: No special storage conditions are required. The substance has a shelf life of 5 years stored under ambient conditions of climatic Zones IV or II, as demonstrated in stability studies performed according to ICH guidelines.

Approved (_X_) Released (_X_) Rejected (___)

This COA is it reproduced from supplier's COA

Supplementary Table S1: Certificate of analysis and composition of beeswax alcohol (BWA) used in the present study.

Supplementary Figure S1

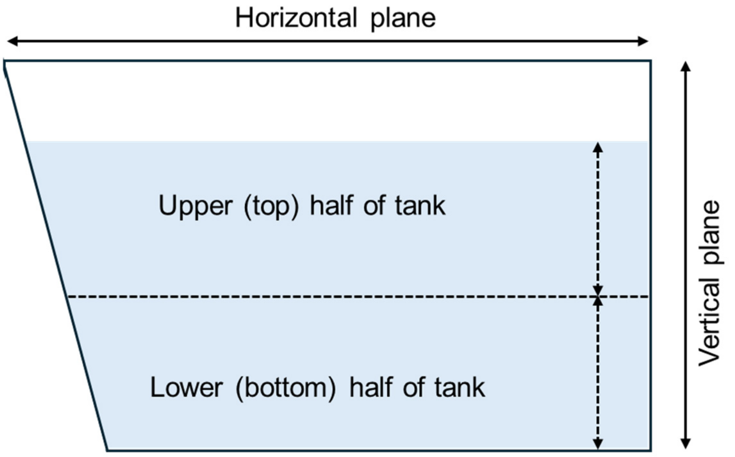

Supplementary Figure S1: Ray diagram of a water tank split horizontally into two sections, with the horizontal line representing the upper (top) and lower (bottom) sections.

Supplementary Method S1

Analysis of plasma

Blood (2 μL) was drawn from the hearts of the adult fish, combined with 3 μL of phosphate-buffered saline (PBS)-ethylenediaminetetraacetic acid (EDTA, final concentration, 1 mM) and then collected in EDTA-treated tubes. The plasma total cholesterol (TC) and triglyceride (TG) were determined using commercial assay kits (cholesterol, T-CHO, and TGs, Cleantech TS-S; Wako Pure Chemical, Osaka, Japan) as per the method suggested by the suppliers. In brief, 5 μL serum was mixed with 200 μL reaction mixture (supplied with a commercial assay kit) for the TC analysis. The content was incubated at 37°C for 10 min, resulting in a red-colored product quantified by adsorption at 490 nm (Microplate reader, Bio-Rad, Hercules, CA, USA).

Similarly, 5 μL serum was mixed with a 200 μL TG-specific reaction mixture (supplied with a commercial assay kit) for TG analysis. The content was incubated for 10 min at 37°C, and the formed colored product was quantified by taking adsorption at 490 nm.

For HDL-C analysis, serum was mixed in an equal ratio with the separation solution (supplied with a commercial assay kit), followed by centrifugation at 3,000 rpm for 10 min. The supernatant (20 μL) was collected and blended with a 200 μL reaction mixture (supplied with a commercial assay kit). After 10 min incubation at 37°C, red color intensity corresponding to HDL-C was quantified by taking absorption at 490 nm.

The commercial diagnostic kit (Asan Pharmaceutical, Hwasung, Republic of Korea) was used to quantify aspartate transaminase (AST) and alanine transaminase (ALT) levels in the serum, following the instructions suggested by the manufacturers. Briefly, 5 μL of serum was combined with 250 μL of either AST or ALT-specific solution, as supplied in the diagnostic kit. Following a 30 min incubation for AST or 60 min incubation of ALT at 37°C, the mixture was then blended with 250 μL of the respective coloring reagent (AST or ATL-specific, provided in the diagnostic kit). After a subsequent 20 min incubation at RT, 250 μL of 0.4 N NaOH was introduced to halt the reaction. Finally, the AST and ATL were quantified by measuring absorbance at 490 nm.
